# Post-operative uric acid: a predictor for 30-days mortality of acute type A aortic dissection repair

**DOI:** 10.1186/s12872-022-02749-9

**Published:** 2022-09-15

**Authors:** Shulun Ma, Qian Xu, Qinghua Hu, Lingjin Huang, Dongkai Wu, Guoqiang Lin, Xuliang Chen, Wanjun Luo

**Affiliations:** Department of Cardiovascular Surgery, Xiangya Hospital, Central South University, Xiangya Rd 87, Changsha, 410008 China

**Keywords:** Type A aortic dissection, Uric acid, Outcomes, Regression analysis

## Abstract

**Background:**

Hyperuricemia is associated with aortic dissection and cardiovascular diseases. The implication of high serum uric acid (UA) level after acute aortic dissection repair remains unknown. The aim of this study is to explore the role of peri-operative serum UA level in predicting 30-days mortality with acute type A aortic dissection (AAAD) patients, who underwent surgery.

**Methods:**

This study retrospectively enrolled 209 consecutive patients with AAAD, who underwent surgery in Xiangya Hospital from 2017 to 2020. Post-operative laboratory examinations were measured within 24 h after surgery. Univariate analysis and logistic regression analysis were used for predictor finding.

**Results:**

209 consecutive AAAD patients were included, 14.3% (n = 30) were dead within 30 days after surgery. By univariate analysis, we found AAAD repair patients with 30-days mortality had a higher prevalence of cerebral malperfusion, lower pre-operative fibrinogen, longer cardiopulmonary bypass and aortic crossclamp time, and higher post-operative day 1 (POD1) creatinine and urea levels. Both pre-operative (433.80 ± 152.59 vs. 373.46 ± 108.31 mmol/L, p = 0.038) and POD1 (559.78 ± 162.23 vs. 391.29 ± 145.19 mmol/L, p < 0.001) UA level were higher in mortality group than in survival group. In regression model, only cerebral malperfusion (OR, 7.938, 95% CI 1.252–50.323; p = 0.028) and POD1 UA level (OR, 2.562; 95% CI 1.635–4.014; p < 0.001) were independent predictors of 30-days mortality in AAAD repair patients. According to the ROC curve, the POD1 UA level provided positive value for 30-days mortality in AAAD repair patients with 0.799 areas under the curve. The optimum cutoff value selected by ROC curve was 500.15 mmol/L, with a sensitivity of 65% and a specificity of 86%.

**Conclusion:**

Pre- and post-operative hyperuricemia are potentially associated with worsened outcomes in AAAD surgery patients. The POD1 UA level has a predictive role in 30-days mortality in AAAD repair patients.

## Introduction

Acute type A aortic dissection (AAAD) is a life-threatening cardiovascular emergency [1, 2]. Despite improved surgical techniques and peri-operative care [3], the short-term mortality rate after aortic dissection (AD) surgery ranges from 15 to 30% in recent years [4–6], especially the 30-days mortality of AAAD, which is 2–3 fold higher than other types of aortic disscetion [6]. Thus, early peri-operative detection, risk of death assessment and timely treatment are essential for improving survival and outcomes of AAAD repair patients.

Hyperuricemia is common in China [7, 8]. Increasing evidence have reported that hyperuricemia is associated with cardiovascular disease including hypertension, coronary artery disease as well as aortic dissection [9–11]. Serum uric acid (UA) is a relatively insoluble end-product of purine metabolism [12] and was found to be increased excreted after cardiac surgery, which may predict risk for cardiac complications and acute renal failure [13]. Although a series of biomarkers including matrix metalloproteinases, TGF-β, creatine kinase, D-dimer, C-reactive protein and sST2 have been reported to serve as possible peri-operative prognostic factor for AAAD [14, 15], the relation between UA and AAAD outcomes remains conflicting [16, 17], and its value for predicting risk and 30-days mortality AAAD repair patients is unreported.

The aim of this study is to explore the role of peri-operative serum UA level in predicting 30-days mortality of AAAD who underwent open repair surgery.

## Patients and methods

We retrospectively enrolled 209 consecutive patients with AAAD, who underwent surgery in our department from January 2017 to October 2020. All patients were diagnosed with AAAD by contrast-enhanced computed tomography and were assessed with transthoracic echocardiography for pericardial effusion and other associated cardiopathy prior to surgery. Patients who refused to receive operation and died before the operation was excluded in our cohort. Standard peri-operative treatments were performed immediately at ICU admission and patients were transferred to emergency surgery within 24 h after admission. Based on the extent of the aortic pathology and experience of the surgeon, four surgical strategies were performed including Sun’s procedure [18], Sun’s procedure with aortic root replacement, ascending aortic replacement with or without hemiarch replacement and modified Bentall procedure. Individuals who died prior to surgery in hospital and patients who received endovascular surgery or diagnosed as AD before were excluded.

Variables included in the study were pre-operative demographic and laboratory data, intra-operative data, post-operative demographic and laboratory data. Medical history, physical and laboratory examinations were acquired at ICU admission. We recorded aortic replacement procedures and time for cardiopulmonary bypass and aortic crossclamp from intra-operative surgery data. Post-operative laboratory examinations were measured within 24 h after surgery.

The primary outcome was 30-days mortality, defined as all-cause death between 0 and 30 days after surgery. The secondary outcome included 30-days complications morbidities, including spinal cord ischemia, stroke, pneumonia, renal failure, delirium, prolonged mechanical ventilation (> 48 h) and myocardial ischemia. These 30-days outcomes were collected from hospital database and follow-up database obtained from surgical clinical nurse reviewers. The definition of all variables, as well as the indication and performance of the surgical procedures are based on the guidelines published by the Society of Thoracic Surgeons definitions [19]. The institutional ethics committee approved the reporting of information obtained from our retrospective study.

Categorical variables were summarized as numbers and frequency (%) and were compared using chi-square test or Fisher Exact test, as appropriate. Continuous variables were summarized as mean ± standard deviation (SD) and compared using Student t test based on normal distribution test results. The risk variables with p < 0.05 in the univariate analysis were included in the logistic regression model, and stepwise elimination method based on binary logistic regression was constructed to identify independent risk factors. Using receiver operating characteristic (ROC) curves, the reliability of the independent risk factors was further evaluated in regard to the 30-days mortality predictor’s sensitivity, specificity and respective areas under the curves (AUCs) with 95% confidence interval (CI). Its optimal cutoff value was determined when sensitivity and specificity reached the peak (The best Youden index). All statistical analyses were performed by using SPSS V 25.0 (SPSS Inc., Chicago, IL).

## Results

Of the 209 AAAD repair patients included in the study, 14.4% (n = 30) were dead within 30 days after surgery. The modality of death included multiple organ dysfunction syndrome (MODS) (n = 11), stroke (n = 7), pericardial tamponade or malignant arrhythmia (n = 4), renal failure (n = 4), infection (n = 3) and aortic rupture (n = 1). Table 1 showed patients with 30-days mortality had higher prevalence of pre-operative cerebral malperfusion (10%) compared to survival group (1.7%) (p = 0.009). No other significant differences were found in clinical demographics such as age, gender, hypertension, diabetes, valvular disease, Marfan syndrome, cerebrovascular disease, ischemic heart disease and chronic renal dysfunction and there were no significant variances in peripheral and cardiac malperfusion (p > 0.05).

**Table 1:** Pre-operative AAAD clinical profile and characteristics

Pre-operative characteristics	30-days Mortality Group (n = 30)	Survival Group (n = 179)	X^2^	p value
*Clinical data*				
Age (y)	51.27 ± 1.94	49.71 ± 0.80		0.468
Male gender	23 (76.7)	126 (70.4)	0.494	0.482
Onset to operation time (h)	74.04 ± 12.06	200.46 ± 153.65		0.419
Hypertension	23 (76.6)	130 (72.6)	0.04	0.841
Diabetes	1 (3.3)	11 (6.1)	0.644	0.422
Valvular disease	3 (10.0)	13 (7.3)	0.08	0.777
Marfan syndrome	3 (10.0)	7 (3.9)	2.176	0.14
CVD	24 (80.0)	141 (78.8)	0.111	0.739
IHD	1 (3.3)	15 (8.4)	1.371	0.242
Chronic renal dysfunction	0	4 (2.2)	0.67	0.412
Malperfusion	8 (26.7)	22 (12.3)	0.454	0.033
Peripheral	2 (6.7)	10 (5.6)	0.084	0.77
Cerebral	3 (10.0)	3 (1.7)	6.764	0.009*
Cardiac	4 (13.3)	9 (5.0)	3.327	0.068

Data are expressed as mean ± SD or n (%)

CVD = cerebrovascular disease; IHD = ischemic heart disease

*p < 0.05 versus survival group

By analyzing intra-operative data (Table 2), we found that aortic replacement procedures did not affect 30-days mortality of the patients (p > 0.05). However, the cardiopulmonary bypass time (CPBT) (232.53 ± 13.31 vs. 191.42 ± 3.97 min, p = 0.006) and aortic crossclamp time (ACCT) (124.77 ± 8.21 vs. 104.76 ± 2.81 min, p = 0.009) were significantly longer in 30-days mortality group than in survival group. For post-operative complications morbidities, 30% (n = 9) patients had brain deficits in 30-days mortality group which is 17-folds higher than survival group (1.7%, n = 3). Similarly, serious complications of vital organs such as heart (36.7% vs. 6.1%, p < 0.001) and kidney (46.7% vs. 7.8%, p < 0.001) occurred more in the mortality group. Apparently, patients who died within 30 days had shorter hospital stay (13.87 ± 2.10 vs. 19.18 ± 0.95 days, p = 0.032).

**Table 2:** Intra-operative and post-operative clinical data

Variables	30-days Mortality Group (n = 30)	Survival Group (n = 179)	X^2^	p value
Replacement procedure			2.256	0.521
Sun	19	111		
Sun + ARR	7	35		
AAR + HAR	2	22		
Modified Bentall	0	8		
CPBT (min)	232.53 ± 13.31	191.42 ± 3.97		0.006*
ACCT (min)	124.77 ± 8.21	104.76 ± 2.81		0.009*
TCA (min)	28.33 ± 1.92	25.16 ± 0.61		0.063
Complications morbidities				
Spinal cord ischemia	2 (6.7)	12 (6.7)	0	0.988
Stroke	9 (30.0)	3 (1.7)	38.864	< 0.001*
Pneumonia	2 (6.7)	27 (15.1)	1.462	0.227
Renal failure	14 (46.7)	14 (7.8)	34.324	< 0.001*
Delirium	4 (13.3)	32 (17.9)	0.331	0.565
Prolonged mechanical ventilation (> 48 h)	3 (10.0)	8 (4.5)	1.649	0.199
Myocardial ischemia	11 (36.7)	11 (6.1)	26.085	< 0.001*
Hospital Stay (d)	13.87 ± 2.10	19.18 ± 0.95		0.032*

Data are expressed as mean ± SD or n (%).

Sun = Sun’s procedure; ARR = aortic root replacement; AAR = ascending aortic replacement; HAR = hemiarch replacement; CPBT = cardiopulmonary bypass time; ACCT = aortic crossclamp time; TCA = total circulatory arrest

*p < 0.05 versus survival group

Table 3 demonstrated pre-operative and post-operative day 1 (POD1) laboratory data from AAAD patients. Non- 30-days mortality related pre-operative risk factors included blood WBC count, RBC count, PLT count, Hb, albumin, DBIL, ALT, AST, urea, creatinine, glucose, PT, APTT, D-Dimer, FDP, CTnl and NT-pro BNP. High level of pre-operative serum UA (433.80 ± 152.59 mmol/L, p = 0.038) and low level of FBG (2.66 ± 1.41 g/L, p = 0.010) were highly associated to 30-days mortality. Additionally, the POD1 UA level in 30-days mortality group was remarkably higher compared to survival group (559.78 ± 162.23 vs. 391.29 ± 145.19 μmol/L, p < 0.001). In addition to POD1 UA level, POD1 urea, creatinine and FBG level were all significant risk factors for 30-days mortality (p < 0.05).

**Table 3:** Pre-operative and post-operative day 1 laboratory variables for 30-days mortality of AAD patients

Variables	30-days Mortality Group (n = 30)	Survival Group (n = 179)	p value
*Pre-operative data*			
WBC count (10^9^/L)	12.66 ± 4.27	11.83 ± 4.08	0.301
Neutrophil/lymphocyte ratio	10.62 ± 6.37	11.00 ± 8.39	0.776
RBC count (10^9^/L)	4.35 ± 0.56	6.83 ± 26.38	0.606
PLT (10^9^/L)	158.23 ± 65.93	172.30 ± 70.85	0.298
Hb (g/L)	129.80 ± 16.80	126.51 ± 20.45	0.390
Albumin (g/L)	39.58 ± 3.55	38.87 ± 4.96	0.432
DBIL (umol/L)	6.83 ± 3.27	7.03 ± 4.08	0.804
ALT (U/L)	147.88 ± 489.86	48.99 ± 124.74	0.281
AST (U/L)	241.18 ± 578.98	63.68 ± 193.05	0.107
Urea (mmol/L)	8.12 ± 2.65	7.34 ± 3.47	0.262
UA (μmol/L)	433.80 ± 152.59	373.46 ± 108.31	0.038*
Creatinine (μmol/L)	139.14 ± 61.42	119.15 ± 75.41	0.157
Glucose (mmol/L)	8.31 ± 2.63	7.77 ± 2.50	0.760
PT (s)	15.47 ± 2.49	14.82 ± 2.80	0.203
APTT (s)	36.07 ± 5.05	36.68 ± 17.32	0.933
D-Dimer (ug/L)	2.42 ± 3.37	2.00 ± 2.81	0.473
FBG (g/L)	2.66 ± 1.41	3.45 ± 2.00	0.010*
FDP (g/L)	26.09 ± 24.27	17.13 ± 20.42	0.123
CTnI (μg/L)	5.95 ± 18.45	0.40 ± 2.79	0.195
NT-pro BNP (ng/L)	1131.14 ± 1145.93	1587.72 ± 3829.33	0.608
*Post-operative day 1 data*			
Urea (mmol/L)	17.89 ± 7.64	14.44 ± 5.29	0.033*
Creatinine (μmol/L)	275.42 ± 104.23	175.06 ± 93.78	< 0.001*
UA (μmol/L)	559.78 ± 162.23	391.29 ± 145.19	< 0.001*
FBG (g/L)	2.50 ± 0.91	2.88 ± 1.24	0.134

Data are expressed as mean ± SD

WBC = white blood cell; RBC = red blood cell; PLT = platelet; Hb = hemoglobin; DBIL = direct bilirubin; ALT = alanine aminotransferase; AST = aspartate transaminase; UA = uric acid; PT = prothrombin time; APTT = partial prothrombin time; FBG = fibrinogen; FDP = fibrin degradation products

*p < 0.05 versus survival group

**Fig. 1:**
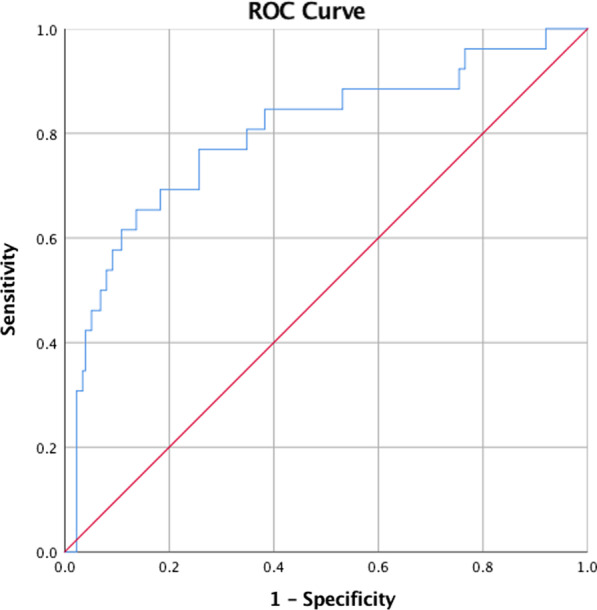
Receiver operating characteristics curve assessing the predictive ability of post-operative day 1 uric acid level in 30-days mortality of AAAD repair patients

Since univariate analyses showed cerebral malperfusion, pre-operative UA, FBG, CPBT, ACCT, POD1 creatinine, urea and UA were risk factors associated with 30-days mortality of AAAD repair. Stepwise elimination method analysis on binary logistic regression were conducted, the results (Table 4) only illustrated cerebral malperfusion and POD1 UA level as independent risk factors for 30-days mortality of AAAD repair, with ORs, (95% CI) of 7.938 (1.252–50.323) and 2.562 (1.635–4.014), respectively. Next, in order to test the predictive value, POD1 UA was subjected to receiver operating characteristic (ROC) curve. As shown in Fig. 1, POD1 UA level provided a positive predictive value with an area under the curve of 0.799. Its optimum cutoff value obtained by ROC curve was 500.15 μmol/L, with a sensitivity of 65% and a specificity of 86%.

**Table 4:** Logistic regression analysis of risk factors for 30-days mortality

Variables	Beta coefficient	Standard error	Odds ratios (95% CI)	p value
Cerebral Malperfusion	2.072	0.942	7.938 (1.252–50.323)	0.028*
POD1 UA (per 1 SD)	0.941	0.229	2.562 (1.635–4.014)	< 0.001*

POD1 UA = post-operative day 1 uric acid

*p < 0.05 in logistic regression analysis

Apparently, the patients that developed post-operative complications were more likely to die within 30-days. To evaluate the association of serum UA level with 30-days complications morbidities outcome, all variables above were constructed into the univariate analysis model. The significant risk variables were listed in Table 5. Compared with the group without complications, the complications group had more ischemic heart disease (IHD) and malperfusion manifestations, underwent longer CPBT, longer prothrombin time and lower FBG. Moreover, patients with post-operative complications had higher admission urea, creatinine, UA and POD1 urea, creatinine, UA level. However, in a multivariate model (Table 6), CPBT and POD1 creatinine but not POD1 UA were the independent risk factors for 30-days complications morbidities, suggesting that the predictive role of POD1 UA in 30-days mortality of AAAD repair patients is independent of the 30-days complications morbidities.

**Table 5:** The risk factors for post-operative 30-days complications morbidities of AAAD repair patients

Variables	30-days complications group (n = 116)	Non-complications group (n = 93)	X^2^	p value
*Clinical data*				
IHD	4(3.4)	12(12.9)	9.401	0.002
Malperfusion	22(19.0)	8(8.6)	4.472	0.034
*Peri-operative data*				
CPBT (min)	208.33 ± 6.14	183.50 ± 4.35		0.009
Hospital stay (d)	20.37 ± 1.38	16.00 ± 0.90		0.002
*Pre-operative laboratory data*				
Urea (mmol/L)	7.98 ± 0.35	6.79 ± 0.27		0.010
UA (μmol/L)	400.85 ± 11.72	358.60 ± 10.49		0.008
Creatinine (μmol/L)	134.32 ± 7.99	106.54 ± 5.30		0.008
PT (s)	15.35 ± 0.31	14.36 ± 0.16		0.006
FBG (g/L)	3.04 ± 0.17	3.71 ± 0.21		0.020
*Post-operative day 1 laboratory data*				
Urea (mmol/L)	16.35 ± 0.59	13.14 ± 0.49		< 0.001
UA (μmol/L)	455.59 ± 16.38	362.72 ± 12.84		< 0.001
Creatinine (μmol/L)	211.33 ± 10.89	148.59 ± 6.65		< 0.001

Data are expressed as mean ± SD or n (%)

CVD = cerebrovascular disease; IHD = ischemic heart disease; CPBT = cardiopulmonary bypass time; UA = uric acid; PT = prothrombin time; FBG = fibrinogen

*p < 0.05 versus survival group

**Table 6:** Logistic regression analysis of risk factors for 30-days complications morbidities

Variables	Beta coefficient	Standard error	Odds ratios (95% CI)	p value
CPBT	0.008	0.004	1.008 (1.000–1.016)	0.043*
POD1 Creatinine	0.014	0.003	1.014 (1.008–1.020)	< 0.001*

CPBT = cardiopulmonary bypass time

*p < 0.05 in logistic regression analysis

## Discussion

In this study, we firstly revealed pre- and post-operative hyperuricemia as risk factors for worsened outcomes in AAAD patients, who underwent surgery. And it is also the very first-time regarding POD1 UA level as a predictor for 30-days mortality of AAAD repair patients, which is independent of the 30-days complications morbidities.

AAAD is a medical emergency that requires acute surgical repair. Despite many improvements in the care of AAAD patients and advanced technical repair procedures, the 30-days mortality after surgery remains very high [20, 21], which makes early peri-operative detecting and crucial treatment implementation become imperative goals for improving AAAD repair short-term mortality. By screening and analyzing all peri-operative data from 209 AAAD repair patients, our study has uncovered pre-operative UA level is a risk factor and POD1 UA level exceeding 500.15 mmol/L may independently predicts 30-days mortality. Peri-operative monitor, care and risk assessment are crucial to aortic dissection patients. Previous studies suggested that various post-operative biomarkers are highly related to adverse outcomes, progress and prognosis for AD patients [22, 23]. From this point, we emphasize the importance of closely monitoring UA of AAAD patients after repair surgery to detect early mortality risk and improve survival outcomes.

In comparison with Western countries, hyperuricemia is common in China [7, 8] and has been reported to be related to many cardiovascular diseases [9–11]. So far, serum UA level has been reported to be associated with adverse outcomes including mortality within the cardiovascular disease population [24]. However, limited research has shown the relationship between UA and AD [25, 26]. One study from Li, X. et al. suggested that increased level of UA found in AD patients might be an independent risk factor of AD [25]. Since renal dysfunction is one of the most common and severe complications of AD [27, 28], renal excretion of serum-insoluble purine metabolite,UA, may be interrupted or even worsen after repair surgery, leading to a sharp rise in UA levels. Furthermore, the elevated level and deposition of UA produced from xanthine oxidase activity represent ROS overproduction and proinflammatory activities, which is an indispensable part in endothelial dysfunction through decreasing nitric oxide production [29–31]. Thus, the endothelial dysfunction and systemic inflammatory activation induced by hyperuricemia may be one explanation of high short-term morality of AAAD repair patients. Here, our novel research revealed POD1 UA level could independently predict 30-days mortality of AAAD patients after surgery, which was not related to 30-days post-operative complications morbidity. Furthermore, our data showed that the level of POD1 UA in patients died from MODS, stroke, pericardial tamponade or malignant arrhythmia, renal failure and infection were 618.71 ± 143.76, 544.76 ± 100.80, 500.87 ± 300.69, 566.42 ± 210.31 and 468.03 ± 157.27 μmol/L, respectively. Despite lack of stastistical significance (p = 0.659) which may cause by limited number in mortality group, our data also indicated that the level of POD1 UA in patients who died from MODS was higher than other patients in mortality group. Endothelial dysfunction is one of the most important contributors to MODS [32]. Elevated POD1 UA may originate from increased xanthine oxidoreductase activity more than renal underexcetion, which might account for acute surgical attack and reperfusion injury after AAAD repair surgery. Higher level of post-operative uric acid might reflect higher inflammatory level and endothelial dysfunction which contributes to the progress of MODS. However, the underlying mechanism needs to be experimentally investigated in the future.

Recent years, a series of biomarkers including matrix metalloproteinases, TGF-β, creatine kinase, D-dimer, C-reactive protein and sST2 have been reported to serve as possible peri-operative prognostic factors for AAAD [14, 15]. Several studies reported biomarkers for 30-days mortality of AAAD patients were inflammatory related molecules such as elevated WBCs, neutrophil–to-lymphocyte ratio and CRP [33–35]. In addition, other laboratory variables including admission D-dimer, creatinine, PLT count, serum lactic acid level [34, 36, 37] were also mentioned of this predictive value. Whereas in our study, no significant differences of WBC count, neutrophil–to-lymphocyte ratio, platelet count, D-dimer, creatinine were observed between 30-days morality group and survival group. The controversial results may differ based upon different clinical institutions and study population. Moreover, the risk factors displayed in our research for 30-days mortality included cerebral malperfusion, CPBT, ACCT, complications of heart, kidney and brain, hospital stay, admission serum UA and FBG level, POD1 urea and creatinine level. In addition to POD1 UA level, cerebral malperfusion was another predictor of 30-days mortality in our patients. These results are in line with other previous studies [27, 28, 38–41], implying our research as credible. Additionally, POD1 UA level as an independent predictor raises the possibility of targeting therapy to reduce or prevent initial increase of UA which may improve short-term outcomes. Some researchers have explored the utility of allopurinol or febuxostat as potential treatments for cardiac complications after sugery [42, 43]. It shed lights on the possibility of utilizing antigout drugs such as allopurinol or febuxostat to reduce hyperuricemia and prevent AAAD 30-days mortality in the future.

There are a few limitations of our study. Firstly, it is a single-center retrospective study with small population. Our results may not be generalized to patients in other centers because patient volume and experiences in peri-operative treatment and surgical intervention of AAAD might be vary depending on clinical centers. In spite of careful statistical design, a retrospective study with limited population is prone to be partially offset. The predictive accuracy of POD1 UA should be validated in a large population, or another cohort and different populations. Finally, the study was developed from data focused only on AAAD patients with surgery. It is unknown whether this conclusion could apply to all types of AD patients or other aorta replacement surgery. In the future, we plan to explore the predictive value of POD1 UA level in a large clinical study containing type B AD patients and patients who have undergone general aortic surgery.

## Conclusion

Pre- and post-operative hyperuricemia are potentially associated with worsened outcomes in AAAD surgery patients. The POD1 UA level has a predictive role in 30-days mortality in AAAD repair patients.

## Data Availability

The data that support the findings of this study are available from the Xiangya Hospital, but restrictions apply to the availability of these data, which were used under license for the current study, are not publicly available. Data are however available from the authors upon reasonable request and with permission of Xiangya Hospital.

## Abbreviations

UA: Uric acid
AAAD: Acute type A aortic dissection
POD1: Post-operative day 1
ROC: Receiver operating characteristic curves
AUC: Areas under the curves
CI: Confidence interval
AD: Aortic dissection
ICU: Intensive care unit
SD: Standard deviation
MODS: Multiple organ dysfunction syndrome
CPBT: Cardiopulmonary bypass time
ACCT: Aortic crossclamp time
WBC: White blood cell
RBC: Red blood cell
PLT: Platelet
Hb: Hemoglobin
DBIL: Direct bilirubin
ALT: Alanine aminotransferase
AST: Aspartate transaminase
PT: Prothrombin time
APTT: Partial prothrombin time
FDP: Fibrin degradation products
FBG: Fibrinogen
CTnl: Cardiac troponin I
BNP: Brain natriuretic peptide
OR: Odd ratio
IHD: Ischemic heart disease
sST2: Soluble ST2
CRP: C-reactive protein
TCA: Total circulatory arrest
